# Impact of docusate and fauna‐free on feed intake, ruminal flora and digestive enzyme activities of sheep

**DOI:** 10.1111/jpn.13382

**Published:** 2020-05-07

**Authors:** Chucai Yu, Qiujiang Luo, Yong Chen, Shimin Liu, Changjiang Zang

**Affiliations:** ^1^ Laboratory of Animal Nutrition College of Animal Science Xinjiang Agricultural University Urumqi China; ^2^ UWA Institute of Agriculture The University of Western Australia Crawley WA Australia

**Keywords:** docusate, enzyme, flora, intake, rumen, sheep

## Abstract

Four Small‐tail Han male hogget sheep, fitted with rumen cannula and fed the same basal diet were used to study the impacts of docusate (DOC) and fauna‐free on the voluntary feed intake (VFI), and ruminal protozoal, bacterial and fungal counts and the digestive enzyme activities. By a 4 × 4 Latin square design, sheep were given no DOC (the control), 2 doses of DOC: 1.2 and 3.0 g/kg diet or oral dose of 6.0 g/d DOC for three days (fauna‐free treatment) in each period of 18 days, the last three days of which were for sampling the rumen fluid. Compared with the control, 1.2 g/kg of DOC supplementation significantly resulted in increases of 18.0% VFI and 44% bacterial count, and no significant change in the fungal number. Supplementing DOC reduced protozoal number in a dose‐dependent manner. The fibre degradation enzyme activity in rumen fluid increased by 17.7% with a concomitant 10% increase in volatile fatty acids (VFA); the protease activity was reduced by 23% with a corresponding reduction in rumen ammonia by 42%. In contrast, supplementing 3.0 g/kg of DOC has adverse effects on those measures compared with 1.2 g/kg of DOC. Defaunation was accompanied with substantial increases in the bacterial and fungal counts, but had no significant influences on VFI and the enzyme activity for starch, protein and pectin digestion, and small changes in fibre digestion enzymes and the total VFA compared with the control. A high correlation (*r* ^2^ = 0.82) was noted between VFI and the total activity of fibre digestion enzymes and VFA. It was proposed that fibre digestion rate in the rumen is a primary factor for determining VFI in sheep, and dietary supplementation of 1.2 g/kg of DOC could partially result in enhanced activity of fibre digestive enzyme in the rumen and increase VFI.

## 1 INTRODUCTION

Docusate (**DOC**), also called as aerosol OT, is a surfactant. Its sodium salt is water‐soluble and can be used as a food additive, emulsifier, dispersant and wetting agent. As a surface‐active chemical, DOC has a toxic effect on the cell membrane of protozoa (Wright & Curtis, 1976). The use of DOC to successfully eliminate ruminal protozoa (defaunation) was firstly reported in cattle by Akkada et al. (1968), and later on in goats (Onodera & Koga, 1987), in rumen fermentation in vitro (Fuchigami, Senshu, & Horiguchi, 1989) and in sheep (Ankrah, Loerch, Kampman, & Dehority, 1990, and recently in our laboratory). Compared to faunated and fauna‐free animals, those reports showed ruminal protozoa had the effects on the digestion of dietary cellulose, starch and protein components, and defaunation alters the rumen ecosystem and tends to lower the rate of ruminal fermentation, although the opposite result was reported in cattle on a high‐grain diet (Nagaraja, Towne, & Beharka, 1992). Protozoa can account for half of the microbial mass in the rumen, engulf bacteria and dietary starch granules, and digest cellulose up to one‐third of dietary fibre; hence, there is an inverse relationship between protozoal and bacterial numbers in the rumen (see Russell & Rychlik, 2001). Because ruminal bacteria play a predominant role in fibre digestion, reducing protozoal number by partial defaunation could be accompanied with an increase in bacteria, then digestion of dietary fibre, which could be particularly significant in roughage‐based diets. This hypothesis has been tested in our laboratory recently.

Feed conversion efficiency, that is the ratio of the product obtained to feed intake, is a key factor to determine profitability of the livestock industry. The use of consumed feed by animals can be simply partitioned into two parts: for the maintenance requirement and the rest for production. Generally, the higher the feed intake, the higher proportion of consumed feed available for production, so the higher feed conversion efficiency. Therefore, increasing voluntary feed intake (VFI) is a significant measure in livestock feeding and management practices. Many factors affect feed intake in ruminants, which has been reviewed numerously, for example, by Allison (1985), Leng, Jessop, and Kanjanapruthipong (1993), and Fisher (2002). Among them, increasing the rates of dietary degradation in the rumen and outflow into the intestine is an effective approach, particularly for roughage‐based diets. In our laboratory, it has been found that the dose of DOC affected VFI of sheep, and the actual effect depended on the DOC dose in the diet. A lower dose of DOC (0.8 g/kg diet) increased VFI of a roughage‐based diet by 30% and 16% for a concentrate‐based diet, whereas a higher dose (4.0 g/kg diet) decreased VFI by 5% (Luo, Li, Chen, Pan, & Zang, 2014); however, the quantity of ruminal protozoa was always decreased with the DOC doses. The other studies showed that supplementation of a low dose of DOC (0.8 g/kg diet) increased the nutrient absorption in the gastrointestinal tract of sheep (Li, Luo, Pan, Zhou, & Keyimu, 2015;Li, Luo, Zang, Yang, & Pan, 2019), resulting in increased carcass weight and carcass lean weight by about 22% and 32% respectively (Li, Luo, Zhou, Li, & Zhong, 2014). On the basis of those results, we proposed that the alteration of rumen microbes in response to a DOC supplementation could be the major mechanism, and the actual effect depends on DOC dosages. In this study, therefore, two doses of DOC, 1.2 and 3 g/kg diet which were confirmed to increase or decrease VFI in sheep in our previous studies, plus 6 g/d DOC to defaunation were examined on their effects on the VFI, rumen protozoal, bacterial and fungal numbers, and activities of digestive enzymes in the rumen. The aim of the research was to define primary factors in rumen fermentation that affect VFI.

## 2 MATERIALS AND METHODS

### 2.1 Experimental animals and design

The use of animals in this study was approved by the Animal Care Committee, Xinjiang Agricultural University (Urumqi, China), and the experimental procedures were in accordance with the University's guidelines for animal research.

Four of Small Tail Han ram, 1 to 2 years old, with body weight of 53.3 ± 3.1 kg, were installed with permanent rumen cannula for this experiment. During the experimental period, the animals with permanent rumen cannula were nursed daily. The animals were fed ad libitum a diet containing mixed concentrates and cornstalk at a ratio of 3:7. Four treatments were applied to those 4 animals over 4 periods in a 4 × 4 Latin Square design. The four treatments consisted of the control (without DOC supplementation), two doses of DOC supplementation at 1.2 and 3.0 g/kg diet, respectively, and 6.0 g/d oral dose of DOC per sheep. For doses 1.2 and 3.0 g/kg treatments, DOC was dissolved in water, then mixed into the concentrate and offered to the animals before the morning feeding. For the large dose of DOC, 6 g/d per sheep, DOC was dissolved in water and injected into the rumen through the cannula before the morning feeding only for the first three days of each period for rumen defaunation. This fauna‐free group was created, with the other groups, to establish a protozoal gradient from zero up to the normal count. The experiment was conducted over four periods, and each period lasted for 18 days, including 15 days for adaptation to the new DOC dose treatment or defaunation, and the following three days for sample collections.

An extra Small Tail Han ram, fitted with a permanent rumen cannula, was fed the same diet without DOC supplementation and used as back‐up sheep to supply its rumen fluid to the experimental sheep to restore their ruminal microbial flora after each period of the treatments. At the end of each period, rumen fluid from the backup sheep was collected and dosed (200 ml/day) into the rumen of each of the experimental sheep for 3 days.

The four replicates per treatment were estimated using the Sample Size Procedure of GenStat (V19, VSN International) on the basis of the residual variance and VFI difference obtained from our previous experiment for Power value of 0.9. The actual Power value for the maximum VFI difference and variance in Table 2 was 0.93.

The powder of DOC, with effective content of 85%, was purchased from Jiashan Jufeng Chemical Co. China.

### 2.2 The feeding and management of animals

The composition and nutrient levels of the basic diet for animals are shown in Table 1. The content of cornstalk in ration was about 70% (i.e. a roughage‐base diet). The analysis of dry matter, organic matter, crude protein, calcium and phosphorus in diet was performed according to AOAC methods (2005). The contents of neutral detergent fibre (NDF), acid detergent fibre (ADF) and acid detergent lignin (ADL) were determined using the procedures of Van Soest, Robertson, and Lweis (1991). The contents of hemicellulose (HC) and cellulose (CEL) were calculated as the differences between NDF and ADF, and between ADF and ADL respectively.

**Table 1:** The composition and nutrient levels of the basic diet (% DM)

Ingredients	Diet composition	Nutrient levels^a^	Contents
Ground cornstalk	68.30	Organic matter	89.88
Corn	19.70	Crude protein	12.84
Cottonseed meal	6.08	Cellulose	26.30
Rapeseed meal	2.02	Hemi‐cellulose	21.60
Premix^b^	3.90	Lignin	3.11
Total	100	Calcium	0.77
		Phosphorus	0.28

^a^ Values were actually measured.

^b^ The premix provides minerals and vitamins per kg of the basal diet: vitamin A 1,350 IU, vitamin D 270 IU, vitamin E 45 IU, iron 16 mg, copper 8 mg, zinc 5 mg, manganese 10 mg, iodine 8.5 mg, cobalt 0.10 mg, selenium 0.20 mg.

The sheep were kept in individual pens. The ration was divided into two equal portions, and fed at 09:00 and 19:00 hr respectively. The concentrate was fed first, and after it was fully consumed, cornstalk was offered. The amounts of feed offered and the residue were recorded daily for calculations of daily feed intake. The amount of feed offered was adjusted daily according to the feed consumption yesterday to allow for 2% ~ 3% of diet residue for measuring VFI. The ratio of roughage to concentrates was maintained constant throughout the whole experimental period. The animals were free access to drinking water.

The fauna‐free sheep was kept in a pen separated from the other sheep to avoid cross‐contamination of ruminal microbes.

### 2.3 Sample collection and pre‐treatment

During the experimental period, the feed samples of mixed concentrate and ground cornstalk were collected every three days, stored and pooled at the end of each period. The samples were grounded through 1‐mm sieve and used for analyses of chemical compositions later on.

After 15 days of adaptation in each period, rumen fluid samples (about 60 ml each) were drawn via the rumen cannula from each sheep before the morning feeding, and then at 1.5, 3, 5, 7 and 10 hr after the feeding for the last three days of each period, so each animal had triplicate samples at each time point. After sampling at 0 hr each day, 4.5 g polyethylene glycol (PEG) 6,000 per sheep, dissolved in about 100 ml water, was injected into the rumen via ruminal cannula for determining the volume and turnover of rumen fluid. The concentration of PEG in each sample of rumen fluid was individually determined. Then, an exponential relationship of the PEG concentration (mg/L, C) with time (h, t) after the feeding was derived as C = C_0_e^−kt^, where C_0_ is the PEG concentration at time of the feeding (*t* = 0), and k is the outflow rate of the rumen fluid (the proportion of the total volume per h). The rumen liquid volume was calculated by dividing the PED dose (4.5 g) with C_0_. The liquid outflow rate (L/h) was then calculated by multiplying the total volume with k value (Hungate, Reich, & Prins, 1971).

The pH of the rumen fluid was immediately measured after sampling. Then, the rumen fluid was filtered through a double layer nylon bag of 40 mesh, and the nylon bag was gently squeezed. After mixing, two aliquots of 5 ml rumen fluid were taken and a drop of saturated mercuric chloride was added. The aliquots were stored at −20℃ for the determination of NH_3_‐N, volatile fatty acids (VFA) and PEG. Two aliquots of 2.5 ml rumen fluid were mixed with an equal volume of 20% formalin and stored at 4℃ for classically microscopic counting of bacteria and protozoa. Another two aliquots of 1 ml rumen fluid were stored in liquid nitrogen for extraction of DNA for measurement of the copy number of fungi. Additional two aliquots of 10 ml rumen fluid were mixed with an equal volume of phosphate buffer (50 mM, pH 6.0) to diluted rumen fluid and stored at −20℃ for the determination of the activities of digestive enzymes. And 5 ml of rumen fluid, with a drop of saturated mercuric chloride, was frozen as a backup sample. Each sample at a given time from an individual animal was stored separately and determined, except for the determination of fungal PCR.

### 2.4 Assays of samples

#### 2.4.1 Microbial counting and fermentation indexes of rumen fluid

The pH of rumen fluid was determined with a pH‐meter (model 510; Cyberscan). NH_3_‐N was measured by magnesium oxide distillation methods from AOAC (2005) and Mohsen, El‐Santiel, Gaafar, El‐Gendy, and El‐Beltagi (2011), and 3.0 ml of rumen fluid was used for each distillation. The concentrations of VFAs were determined by gas chromatography (Hamada, Omori, Kameoka, Horii, & Morimoto, 1968), and 0.1 ml of rumen fluid was used for each test. The method for PEG measurement was referenced to Hyden (1961) as described, and 1.0 ml of rumen fluid was used. The methods of microscope counting and classification of rumen bacteria and protozoa were according to Chung and Hungate (1976) and Dehority (1984), but because of the difficulty of distinguishing between Coccus and Streptococcus, the two bacteria were counted and pooled as one index.

The triplicate rumen fluid samples at each sampling time, with the same volume, were mixed, and 1 ml was taken for extraction of DNA by the cationic detergent cetyltrimethylammonium bromide (CTAB) and physical crushing method (Minas, McEwan, Newbold, & Scott, 2011). The concentration of DNA was determined by optical reading in a microplate reader. Then, the sample was diluted with TE buffer (Tris‐HCl 10.0 mM, EDTA 1.0 mM, pH 8.0). The sample was then PCR‐amplified following the primers of fungal conserved regions (Denman & McSweeney, 2006), and the fungal copy numbers were measured by real‐time fluorescent quantitative PCR determination (Lwin, Hayakawa, Ban‐Tokuda, & Matsui, 2011). Each sample was assayed in duplicate, and the average value was used.

#### 2.4.2 Digestive enzyme activities in rumen fluid

The enzyme activities of endocellulase, exocellulase, cellobiose, xylanase, pectinase, amylase and protease in the rumen fluid were determined.

Endocellulase activity was determined according to Agarwal, Kamra, and Chaudhary (2000), and the substrate for the endocellulase activity was 0.5% sodium carboxymethyl cellulose (CMC‐Na) solution. Briefly, 0.5 g of CMC‐Na was dissolved in 100 ml of 50 Mm, pH6.0 phosphate buffer. One millilitre of the substrate solution was added to 10‐mL graduated test tube and was incubated at 39 ℃ in a water bath for 30 min with oscillation. Then, 1 ml of warmed rumen fluid was added and incubated for exactly 3 min. Then, 2 ml of dinitrosalicylic acid (DNS) was added to stop the reaction. Samples were then incubated in boiling water bath for 10 min, followed with immediately cooling under running water. Samples were then topped up to exact 10 ml volume with distilled water, shaken, transferred into 10‐mL centrifugal tubes and centrifuged at 1,500 × g for 10 min. The supernatant was used for the assay at OD_550_ (Lee et al., 2003).

The substrate for determination of the exocellulase activity was 0.5% microcrystalline cellulose suspension (Agarwal et al., 2000) which was prepared as followings: 0.5 g ash‐free filter paper was cut into tiny pieces and placed into 200‐ml beaker flask with 50 mM phosphate buffer (pH 6.0), topped to 100 ml volume. After adding glass beads, the flask was shaken on a horizontal shaker for 48 hr. The determination procedure was the same as that for the endocellulase activity, except that the amount of the substrate and the diluted rumen fluid were 1 ml and 0.6 ml respectively.

The substrates for the activities of cellobiase (Peiji, 1987), xylanase (Agarwal et al., 2000) and pectinase (Miller, 1959) were 0.5% salicin, 0.5% xylan and 0.5% pectin respectively. The determination procedures were the same as described for the endocellulase activity assay, and the volume of the substrates was 1 ml for all, and the diluted rumen fluid was 1 ml, 0.5 ml and 0.5 ml respectively (Peiji, 1987).

The substrate for the amylase activity was 0.5 ml of 1% starch. The substrate was mixed with 60 μL rumen fluid and incubated at 39℃ for 3 min. The reaction was stopped by adding 1.0 ml DNS and then bathed in boiling water for 10 min. After cooling under running water, the volume was topped exactly to 100 ml with distilled water. The value at OD_540_ was determined (Engvall, 1980).

For the determination of the protease activity, 1.0 ml of rumen fluid and 1.0 ml of 2% casein were pre‐heated at 39℃ in water bath for 2 min, mixed and incubated for 3 min. After an addition of 2 ml of 0.4 M trichloroacetic acid, the solution was incubated at 39℃ in water bath for 20 min and then centrifuged at 1,800 × g for 10 min. One millilitre of the supernatant, 5.0 ml of 0.4 M sodium carbonate and 1.0 ml of diluted Folin reagent were mixed thoroughly and then incubated at 39℃ for 20 min. The value at OD_660_ was determined (Brock, Forsberg, & Buchanan‐Smith, 1982).

The procedures for the standard curves for various enzyme activities were the same as described in the sample assays. The standard substances for the enzyme activity assays were glucose for endocellulase, exocellulase and cellobiase; xylose for xylanase; galacturonic acid for pectinase; maltose for amylase; and tyrosine for protease respectively. The spectrometer used for recording the OD values in above assays was a Shimadzu UV 1,800 (Shimadzu [China])

### 2.5 Statistical analysis

The data were subjected to the analysis of variance for a 4 × 4 Latin square design using the general linear models (GLM) of SPSS 18.0 statistical software (IBM). The model was yijk = *μ* + *α_i_* + *β_j_* + *γ_k_* + *ε_ijk_*, where *y_ijk_* is an observation,* μ* is the overall mean, *α* is the fixed effect of DOC supplementation (*j* = 1–4), *β* is the fixed effect of animal (*i* = 1–4), *γ* is the fixed effect of treatment periods (*k* = 1–4); *ε_ijk_* is the residual error. Since the rumen fluid samples were collected at different times after the feeding and analysed, the average of the measures across the times was calculated and used for statistical analyses. Those mean values and the standard error of the means (*SEM*) are presented unless otherwise stated for the notable time‐related patterns. Statistical significance was declared at a level of *p* ≤ .05. Multiple comparisons among the treatment means were performed by Duncan's New Multiple Range Test.

## 3 RESULTS

### 3.1 VFI

As shown in Table 2, VFI reached the highest when DOC was supplemented at 1.2 g/kg among four groups (*p* ≤ .05), and DM intake was increased by 18.0% compared with that in the control. There was no significant difference in VFI between the control, 3.0 g/kg DOC and fauna‐free groups (*p* > .05).

No significant changes in the volume (5.53 L, *SEM* 0.08) and turnover rate (0.40 L/h, *SEM* 0.02) of the rumen fluid of sheep were found among the four groups (*p* > .05), suggesting the DOC supplementation has no effect on the turnover of the rumen fluid, so the comparison of the measures in the fluid based on a volume unit was acceptable.

**Table 2:** The effects of two doses of docusate and fauna‐free on the voluntary intake of sheep (g/day, *n* = 4)

DOC g/kg diet DM	0	1.2	3	FF	*SEM*	*P‐*value
Dry matter	930.0^b^	1,097.2^a^	867.6^b^	898.0^b^	34.06	0.012
Organic matter	874.8^b^	1,032.2^a^	817.9^b^	844.7^b^	30.51	0.010
Crude protein	119.4^b^	140.9^a^	111.4^b^	115.3^b^	4.37	0.012
Mixed concentrate	275.2^b^	317.4^a^	263.5^b^	265.4^b^	11.15	0.043
Cornstalk	654.8^b^	779.8^a^	604.1^b^	632.6^b^	20.40	0.004

Abbreviations: DOC, docusate; FF, fauna‐free (6 g/d DOC per sheep); *SEM*, standard error of the mean.

^a,b,c^ Means with different superscript letters within a row differ significantly (*p* ≤ .05).

### 3.2 pH, HN_3_‐N, VFAs and enzyme activity in rumen fluid

The pH, HN_3_‐N and VFA concentrations, and enzyme activity in rumen fluid are shown in Table 3. Compared with those in the control, the pH value was significantly lower at the 1.2 g/kg DOC dose (*p* ≤ .05), but higher at 3.0 g/kg DOC dose; the ammonia concentration was significantly lowered at both 1.2 and 3.0 g/kg DOC doses (*p* ≤ .05) in a dose‐dependent manner; the total VFA was significantly higher at 1.2 g/kg DOC dose (*p* ≤ .05), but lower at 3.0 g/kg DOC dose (*p* ≤ .05), primarily attributed to the corresponding changes in acetate (*p* ≤ .05) and butyrate (*p* ≤ .05). Defaunation by dosing 6.0 g/d DOC resulted in no significant change in the pH value (*p* ≤ .05), reduction in the ammonia concentration and little decreases in acetate, propionate and butyrate concentrations, which added up a significant reduction in the total VFA (*p* ≤ .05) compared with those in the control.

**Table 3:** The effects of two doses of docusate and fauna‐free on pH, NH_3_‐N (mg/100ml), VFAs (mmol/L) and enzyme activities (U/mL) in rumen fluid of sheep (*n* = 4)

DOC g/kg diet DM	0	1.2	3.0	FF	*SEM*	*P‐*value
pH	6.49^b^	6.38^c^	6.58^a^	6.45^bc^	0.02	.006
NH_3_‐N	21.21^a^	12.36^b^	6.60^d^	10.56^c^	0.03	<.001
Acetic acid	64.00^b^	70.56^a^	46.83^c^	62.92^b^	0.34	<.001
Propionic acid	13.91^a^	14.30^a^	10.89^b^	13.34^a^	0.30	.001
Butyric acid	7.10^b^	8.63^a^	4.26^c^	6.96^b^	0.21	<.001
Total VFA	85.14^b^	93.50^a^	61.98^d^	83.31^c^	0.35	<.001
Endocellulase	24.46^b^	29.07^a^	18.49^d^	23.67^c^	0.23	<.001
Exocellulase	25.18^b^	28.85^a^	19.61^c^	24.72^b^	0.17	<.001
Cellobiase	11.89^b^	14.50^a^	8.62^d^	10.57^c^	0.13	<.001
Xylanase	171.69^b^	170.57^bc^	169.85^c^	175.61^a^	0.50	.001
Pectinase	131.75^a^	124.45^c^	125.90^bc^	130.01^ab^	1.42	.031
Amylase	29.60	30.32	28.87	26.86	0.85	.112
Protease	5.73^a^	4.43^b^	3.49^c^	5.63^a^	0.10	<.001

Note

Each value is the arithmetic mean of 4 sheep, and each had 18 measures (6 times/d × 3 d).

Means with different superscript letters within a row differ significantly ((*p* ≤ .05).

Abbreviations: DOC, docusate; FF, fauna‐free (6 g/d DOC per sheep); *SEM*, standard error of the mean; VFA, volatile fatty acid.

As shown in Table 3, compared with the control, the 1.2 g/kg DOC dose significantly enhanced the activities of cellulase (17.7%), namely endocellulase (18.8%), exocellulase (14.6%) and cellobiase (22.0%), respectively, resulted in no significant changes in the xylanase and amylase activities (*p* > .05), but decreased the pectinase and protease activities (*p* ≤ .05); the 3.0 g/kg DOC dose significantly reduced the activities of all enzymes (*p* ≤ .05) except for amylase (no significant change, *p* > .05). Defaunation by dosing 6.0 g/d DOC resulted in variable changes in the enzyme activity: decreases in endocellulase and cellobiase (*p* ≤ .05), increases in xylanase (*p* ≤ .05), while no significant changes in exocellulase, pectinase, amylase and protease (*p* > .05) compared with control.

### 3.3 Micro‐organisms in rumen fluid

The numbers of protozoa, Isotricha, Entodinium, Diplodinium, and the total of them in rumen fluid, as shown in Table 4, were reduced significantly (*p* ≤ .05) in a dose‐dependent manner by the additions of DOC. Compared with the control (Table 4), the total number of rumen bacteria was significantly increased (*p* ≤ .05) at the 1.2 g/kg DOC dose, composed of the substantial increases in the numbers of Small bacilli, Coccus + Streptococcus and Selenomonas, and a decrease in Big bacilli (*p* ≤ .05 for all); the 3.0 g/kg DOC dose significantly reduced the total number of rumen bacteria: the numbers of Big bacilli, Small bacilli, Coccus + Streptococcus and Selenomonas all reduced (*p* ≤ .05 for all), except for an increase in the Selenomonas number (*p* ≤ .05). Defaunation by dosing 6 g/d DOC dramatically increased the total bacterial number and the numbers of Big bacilli, Small bacilli, Coccus + Streptococcus and Selenomonas (*p* ≤ .05 for all), except for a decrease in the Selenomonas number (*p* ≤ .05) compared with the control.

**Table 4:** The effects of two doses of docusate and fauna‐free on the numbers of protozoan (×10^5^/ml), bacteria (×10^9^/ml) and DNA copies of fungi (×10^7^copies/mL) in rumen fluid of sheep (*n* = 4)

DOC g/kg diet DM	0	1.2	3.0	FF	*SEM*	*P‐*value
Isotricha	1.02^a^	0.45^b^	0.07^c^	0^d^	0.01	<.001
Entodinium	10.48^a^	4.58^b^	1.43^c^	0^d^	0.10	<.001
Diplodinium	0.62^a^	0.23^b^	0.18^c^	0^d^	0.01	<.001
Total protozoa	12.11^a^	5.26^b^	1.67^c^	0^d^	0.11	<.001
Big bacilli	0.56^a^	0.25^c^	0.08^d^	0.37^b^	0.01	<.001
Small bacilli	7.74^c^	18.50^b^	4.50^d^	22.63^a^	0.07	<.001
Coccus + Streptococcus	117.00^c^	162.59^b^	86.95^d^	189.82^a^	1.87	<.001
Selenomonas	1.11^c^	1.15^b^	1.67^a^	0.54^d^	0.01	<.001
Total bacteria	126.40^c^	182.51^b^	92.74^d^	213.35^a^	1.21	<.001
Fungi	5.80^bc^	6.74^ab^	4.87^c^	7.52^a^	0.44	.023

Note

Each value is the arithmetic mean of 4 sheep, and each had 18 measures (6 times/d × 3 d).

Means with different superscript letters within a row differ significantly (*p* ≤ .05).

Abbreviations: DOC, docusate; FF, fauna‐free (6g/d DOC per sheep); *SEM*, standard error of the mean.

The fungal count, assayed as the DNA copies, in the rumen fluid shown in Table 4 was significantly increased (*p* ≤ .05) both at the 1.2 g/kg DOC dose and defaunation by dosing 6.0 g/d DOC, whereas the 3.0 g/kg DOCs dose had no significant influence on the fungal number (*p* ≤ .05), when compared with the control.

## 4 DISCUSSION

The primary findings of the present study are as follows: (a) ruminal protozoa could be partially moved by dietary supplementation of DOC; (b) supplementation of DOC could increase VFI in sheep; (c) the effect of DOC supplementation on VFI depended on the DOC dosage; and (d) an effective dose of DOC for increasing VFI was at 1.2 g/kg diet in this experiment.

The primary mechanism of DOC supplementation in sheep was on the significant changes in the composition of rumen microbes: the protozoal number was consistently reduced with the increase in the DOC doses, and fully eliminated at DOC dose 6 g/d; the concomitant changes in the bacterial and fungal counts varied depending on the DOC dose: 69% increase in bacteria in fauna‐free group, 44% increase at 1.2 g/kg DOC dose, but 27% decrease at 3.0 g/kg DOC dose, while the changes in the fungal count with DOC dose had a similar trend to that of bacteria. The close relationship between the protozoal number and DOC dosage indicates DOC has a direct and toxic role in the life of protozoa in the rumen. The findings support our hypothesis that the alteration of rumen micro‐organisms in response to a DOC supplementation is the major mechanism, and the actual effect is associated with the DOC dosage. This result is consistent with our previous study on the effect of DOC on VFI in sheep (Luo et al., 2014).

To understanding interplay relationships between the rumen microbes and the fermentation indices (e.g., VFAs, ammonia, enzyme activities) in the rumen fluid, we performed correlation analyses between them and presented the results in Table 5. As shown in Table 5, although the 56.6% decrease in protozoa was associated with the 18.9% increase in VFI at DOC dose 1.2 g/kg diet, the further decrease in protozoa at DOC dose 3.0 g/kg and defaunation treatments actually reduced VFI, albeit not statistically significant, suggesting that the change in the protozoal number in the rumen was not only the reason for the changed voluntary intake, rather the concomitant changes in protozoa, bacteria and fungi determined the intake. Those changes in the bacterial and fungal counts were significantly correlated with the total activity of endocellulase, exocellulase, cellobiase and xylanase (*r* ^2^ = 0.59). The reported effect of ruminal protozoa on dietary degradation in the rumen is also controversial (Jouany, 1996;Park, Yang, & Yu, 2019;Veira, 1986). It has been noted in literature that when the protozoa number decreases, the number of bacteria in the rumen increases significantly (Ozutsumi, Tajima, Takenaka, & Itabashi, 2006;Park et al., 2019). In this experiment, a similar relationship was noted between the protozoal and bacterial numbers, except that the bacterial number was substantially reduced at DOC dose 3.0 g/kg. The reason was unknown. The VFI was highly correlated with the total activity of cellulase degradation‐related enzymes, namely endocellulase, exocellulase and cellobiase (*r* ^2^ = 0.83), followed with a moderate correlation (*r* ^2^ = 0.43) with the amylase activity. As a result of these enzyme activity changes, the total VFA and individual VFAs (acetate, propionate and butyrate) were also highly correlated with VFI. The protease activity was highly correlated with the ammonia concentration in rumen fluid, but not correlated with VFI. Overall, we proposed that supplementing an appropriate dose of DOC can reduce protozoa population, and increase bacteria and fungi in the rumen, which results in an increase in dietary fibre degradation and VFI.

**Table 5:** The correlation between the voluntary feed intake of sheep and the rumen microbes, fermentation metabolites and digestive enzymes (% of increased or decreased, compared with the control)

DOC g/kg diet DM	0	1.2	3.0	FF	Person Correlations (r)	*P‐*value
Voluntary intake change (% of control)	0	18.0	−6.7	−3.4		
Protozoa (total)	0	−56.6	−86.2	−100	0.181	.503
Isotricha	0	−55.9	−93.1	−100	0.208	.440
Entodinium	0	−56.3	−86.4	−100	0.186	.492
Diplodinium	0	−62.9	−71	−100	0.094	.730
Bacteria (total)	0	44.4	−26.6	68.8	0.320	.226
Big bacilli	0	−55.4	−85.7	−33.9	0.085	.755
Small bacilli	0	139	−41.9	192.4	0.258	.334
Coccus + Streptococcus	0	39	−25.7	62.2	0.342	.194
Selenomonas	0	3.6	50.5	−51.4	0.012	.964
Copies of fungi	0	16.2	−16.0	29.7	−0.383	.143
pH	0	−1.7	1.4	−0.6	0.300	.258
NH_3_‐*N*	0	−41.7	−68.9	−50.2	0.140	.605
Total VFA	0	9.8	−27.2	−2.1	0.703	.002
Acetic acid	0	10.3	−26.8	−1.7	0.741	.001
Propionic acid	0	2.8	−21.7	−4.1	0.805	<.001
Butyric acid	0	21.5	−40.0	−2	0.696	.003
Endocellulase	0	18.8	−24.4	−3.2	0.654	.006
Exocellulase	0	14.6	−22.1	−1.8	0.673	.004
Cellobiase	0	22.0	−27.5	−11.1	0.265	.321
Xylanase	0	−0.7	−1.1	2.3	0.416	.109
Pectinase	0	−5.5	−4.4	−1.3	−0.283	.289
Amylase	0	2.4	−2.5	−9.3	0.566	.022
Protease	0	−22.7	−39.1	−1.7	0.270	.312

Abbreviations: DOC, docusate; FF, fauna‐free (6 g/d DOC per sheep); VFA, volatile fatty acid.

Both rumen protozoa (Belzecki, Miltko, Kwiatkowska, & Michalowski, 2013;Miltko et al., 2015) and bacteria (Lokapirnasari, Nazar, Nurhajati, Supranianondo, & Yulianto, 2015;Zorec, Vodovnik, & Marinšeklogar, 2014) produce cellulases. The present study showed that the total cellulase activity in the rumen of sheep without DOC supplementation and in fauna‐free sheep were relatively lower than those sheep supplemented with 1.2 g/kg DOC. High activities of cellulases enhance the fibre degradation rate in the rumen, which may empty the rumen faster and stimulate feed consumption of the host. However, we did not note any significant changes in the rumen liquid volume and the outflow rate in the current study. The outflow rate of particles might be changed, which needs a further study. Our previous studies have shown that the effect of DOC on increasing VFI was much stronger in sheep fed a roughage‐based diet (30.7%) than a concentrate‐based diet (16.3%) (Luo et al., 2014), suggesting that fast digestion of fibre in the rumen can increase VFI, which is in line with the result of the current study (68% roughage). Thus, it is presumed that adding exogenous cellulase would increase VFI of ruminants, which needs to be tested in further studies.

It was noted in the present study that supplementing DOC at 3.0 g/kg diet removed 86% of ruminal protozoa, while the bacterial and fungal counts were reduced 27% and 16%, respectively; correspondingly the digestive enzyme activities (except for amylase) declined, and the total VAF and ammonia in the rumen fluid reduced substantially. Notably, the suppression of the ruminal fermentation was due to the reduced microbes. The result of this treatment is conflict with the general conclusion that the bacterial number is reversely correlated with the protozoal number in the rumen. We have no clear explanation for this effect, but suspect that DOC, as a surfactant, might have a detectable toxic effect on ruminal bacteria when its concentration reaches a threshold, for example, sulphonate detergents can impair the structure of anaerobic microbial membrane in anaerobic fermentation of kitchen wastewater, and the 50% inhibitory concentration of the detergent was about 600 mg/L (Lee, Park, Khanal, & Lee, 2013). In the current experiment, the rumen liquid volume in sheep was about 5.3 L, and the daily consumption of DOC was about 2.6 g at 3.0 g/kg diet, so the estimated DOC concentration in the rumen may be 470 mg/L, which may have toxic effects on ruminal bacteria and needs to be further studied. In addition, the DOC supplementation in this study lasted only for 18 days. Considering the adaptability of rumen microbes to dietary treatments and supplementation, further long‐term investigations are warranted on the effect of DOC on partial defaunation and rumen fermentation in ruminants.

Because of the positive effects of an appropriate DOC dose on VFI and digestive activities of cellulases in the rumen, nutrient absorption in the gastrointestinal tract, and the carcass weight and lean tissue weight of sheep, DOC could be useful in sheep farming.

## 5 CONCLUSION

It was concluded that supplementing 1.2 g/kg DOC in diet effectively reduced the protozoal number, while increased the total bacterial and fungal counts in the rumen of sheep. These changes enhanced the fibre digestive enzymes, which in turn resulted in an increase in feed intake in sheep. Supplementing 3.0 g/kg DOC had adverse effects. Dosing 6.0 g/d DOC eliminated protozoa in the rumen and had no significant influence on feed intake. It is proposed that fibre digestion rate in the rumen is a primary factor for determining VFI in sheep, and partial defaunation may be considered for animals on a roughage‐based diet.

## CONFLICT OF INTEREST

We certify that there is no conflict of interest with any financial organization regarding the material discussed in the manuscript.

## ETHICAL STATEMENT

The authors confirm that the ethical policies of the journal, as noted on the journal's author guidelines page, have been adhered to and the appropriate ethical review committee approval has been received. The authors confirm that they have followed the EU standards for the protection of animals used for scientific purposes.

## ANIMAL WELFARE STATEMENT

The use of animals and the experimental procedures in this study were approved by the Animal Care Committee, Xinjiang Agricultural University (Urumqi, China). All animals involved in this experiment were inspected and cared routinely by a registered veterinarian to ensure all the animals in healthy status. The trial was completely non‐invasive.
